# Comparative thermo-stability of two Rift Valley fever virus vaccine candidate CL13T with a recombinant arMP-12ΔNSm21/384

**DOI:** 10.6026/97320630016547

**Published:** 2020-07-31

**Authors:** S Daouam, Z Boumart, A Elarkam, J Hamdi, KO Tadlaoui, MM Ennaji, MEL harraka

**Affiliations:** 1 Research and Development Virology, Multi-Chemical Industry, Morocco; 2 Laboratory of Virology, Microbiology, Quality and Biotechnology/ETB, Faculty of Sciences and Technics Mohammedia, Morocco

**Keywords:** stability, Clone 13T vaccine, arMP-12ΔNSm21/384 vaccine

## Abstract

Rift Valley fever (RVF) is a zoonotic, viral disease, transmitted by mosquitoes, characterized by high mortality rates in young animals. RVF is an endemic and enzootic disease in the Arabian Peninsula and Africa, causing public health and economic instability. Therefore, it is important to develop vaccines to minimize outbreaks and combat the disease. We documented the stability of the thermo-stability of live attenuated RVF CL13T and recombinant arMP-12ΔNSm21/384 vaccine candidates at different temperatures, including these vaccine viruses in liquid and lyophilized form. The study revealed that both CL13T and recombinant arMP-12ΔNSm21/384 strains were stable for more than 18 months at 4°C. We show that at room temperatures (37°C and 45°C) the CL13T was less temperature sensitive than MP-12NSm-del in both lyophilized and liquid form. These findings are useful for the preparation of RVF vaccines that will avoid the need for a cold chain and therefore, will improve the application of the vaccines under field conditions.

## Background

Rift Valley fever virus (RVFV) is a member of the order Bunyavirales, family Phenuiviridae, genus Phlebovirus [1,2], the virus causes a threat to human and animal health, and significant economic loss. The disease is endemic and enzootic in Africa and the Arabian Peninsula. Outbreaks caused by the RVF have led to restrictions on the movement and slaughter of animals in the affected countries [1]. The disease is most severe in ruminants, causing abortions in pregnant females and high mortality in young animals, (sheep and cattle), that serve as amplifying hosts for the virus and are a link between humans and competent mosquito vectors [2]. Besides, every human living in close contact with their livestock is at risk of infection from possible exposure to blood, products of abortion or tissues from viremic animals [3].

Vaccination is considered an effective strategy against RVF. Several vaccines have been developed. The available live attenuated Smithburn vaccine that originated from South Africa, have been used in Africa countries and Saudi Arabia, however, this vaccine cause abortions and teratogenic effects [3]. The RVFV MP-12 vaccine was promoted as on alternative to the RVF Smithburn vaccine for both human and veterinary use. This vaccine was shown safe and immunogenic in newborn and did not induce abortion in pregnant cows, however one report claimed that the vaccine cause teratogenic effect in pregnant ewes [4]. Another alternative vaccine has been used in livestock, the RVFV Clone 13 (C13) vaccine. This vaccine is a natural attenuated mutant isolated in the Central African Republic from a patient with clinical RVF, and the C13 strain had a natural deletion of 70% of its NSs gene [5]. Clone 13 vaccine has been showed to be safe and immunogenic in pregnant sheep, goat and calves, however, a recent study report that the vaccine could cross the ovine placental and cause fetal infections and malformation [4]. Another study showed that the C13 vaccine was temperature sensitive limiting its use in tropical countries. Effort to improve clone 13 (CL13) stability was recently reported [6]. The recombinant arMP-12ΔNSm21/384 vaccine strain is safe immunogenic and confer a satisfactory immune response in domestic ruminants [7].With the continuing outbreaks of RVF, with the more recent outbreak in livestock in Western [8-9] and Eastern Africa [10], there is an urgent need for a thermostable stable vaccine. Therefore, the objective of this study was to assess the temperature sensitivity of two attenuated RVFV vaccine strains; clone 13T and recombinant arMP-12ΔNSm21/384. The storage and the use of the vaccine at different temperatures under field conditions in the tropics represent a major threat to the efficiency of a vaccination campaign.

## Materials and Methods:

### Cells and Viruses:

The thermo-stable RVFV Clone 13T vaccine (CL13T) was derived by cloning the CL13 virus that was isolated from a human case as a naturally attenuated strain and was rendered thermostable by three cycles of heating (56°C) and selection of thermotolerant particles. The resulting candidate vaccine (CL13T) was stable at 4°C for 20 months and shows significantly improved levels of thermostability over the existing the CL13 vaccine when tested after exposure to different temperatures [11]. A reverse reverse genetic system was used to recover the RVFV MP-12 vaccine strain for the development of the RVFV arMP-12ΔNSm21/384 vaccine, which lacks NSm gene at the pre-Gn region in the M segment and retains the independent attenuating mutations of both the L and M segments [12]. Using the same method described by Calpen et al, we generated arMP-12ΔNSm21/384 for use as a new master virus arMP-12ΔNSm21/384 that was produced and characterized by sequencing the full genome, the potential reversion to virulence, the safety and immunogenicity has been tested in animals [13].

The two vaccine viruses were produced separately on Vero cells maintained in Dulbecco's modified minimum essential medium (DMEM) containing 10% fetal bovine serum (FBS). The culture medium from the Vero cells was removed and replaced with an inoculum of RVFV CL13T or arMP-12ΔNSm21/384 at a multiplicity of infection (MOI) of 0.01. After 1 hour of incubation at 37°C, the inoculum was removed and replaced with 10 mL of DMEM with 10% FBS and incubated at 37°C for 2 to 3 days until cytopathic effect (CPE) became apparent. When cells showed CPE, the supernatant was harvested and formulated by mixing V/V the viral suspension of CL13T or arMP-12ΔNSm21/384 with a stabilizer of lyophilization (4 % peptone, 8 % sucrose and 2 % glutamate).

### Stability study:

The two freeze-dried RVFV vaccine were tested for stability at 4°C for 18 months and at an accelerated process by repeated titrations to determine infectivity titers after exposure in water bath temperatures of 24°C, 37°lC and 45°C, with aliquots of virus titrated every 3 days for 15 days and daily for 4 days respectively. After the reconstitution of the freeze-dried vaccine viruses in the diluent (saline solution),aliquots in the glass vials were prepared and stored in incubators at 24°C, 37°C and in a water bath at 45°C and then tested by periodic titration to determine infectivity titers:

[1] At 4°C and 24°C, with aliquots of virus titrated at 2, 4, 7 and 10 days post exposure.

[2] At 37°C, with aliquots of virus titrated at 6, 12, 18, 24 and 32 hours post exposure.

[3] At 45°C, with aliquots titrated for 2, 4, 6, 12, 14 and 24 hours post exposure.

The thermal stability evaluation of the two vaccines CL13T and arMP-12ΔNSm21/384 was repeated 3 times under the same conditions as used in the first or should it be in these experiments. The average of the 3 infectivity titers was calculated at each exposure time.

Tissue Culture Infectious Dose 50% Endpoint (TCID50) Titration Method.

### Preparation of suspension cells:

The TCID50 endpoint infectivity titration method was used in this study [14]. The vero cells were maintained in 75cm2 flask using DMEM growth medium that contained 10% of FBS and examined daily under a microscope. Vero cell monolayers 3 to 4 days at 90% confluency was placed under a laminar hood to trypsinize. The medium was discarded into a waste beaker and then the cells were washed twice with phosphate buffered saline (PBS). Five mL of trypsin (0.25% trypsin - 0.1% EDTA) was then added and the cells were incubated for 2 to 3 minutes at 37°C. Once the cells where detached, the trypsin was neutralized by adding 10 mL of the cell growth medium containing with 10% of FBS. A volume of 100 µL of the cell suspension was used to perform the cell counting, and then a concentration of 120,000 cells per mL was prepared and used to seed flask for use in the experiments to determine infectivity titers.

### Virus dilution:

The infectivity titers of the freeze-dried RVFV vaccine viruses were was determined by titration on Vero cells using the TCID50 method. The contents of two vials of the reconstituted freeze-dried vaccine viruses were diluted 10-fold diluted until 10-3 and 100 µl of each dilution was added to wells of 96-well plates, 6 wells per dilution. Virus dilution was carried out on ice. Then, 150 µL of Vero cell suspension were added to each well (120000 cell/mL) and the cells and inoculum were incubated at 37°C, 5% CO2 for 5 to 7 days.

### Expression of the results and calculation of the titer:

The cells were examined for cytopathic effect (CPE), and the cells with a CPE were considered positive and those without CPE were considered negative. The titer of the each vaccine virus was determined using the Reed-Muench method [14].

### Statistical Analysis:

The infectivity titer of freeze-dried and liquid form at different temperature of storage was compared between the RVFV CL13T and arMP-12ΔNSm21/384 vaccine viruses using the t-test. A p-value ≤ 0.05 was statistically significant.

## Results:

**Figure 1:**
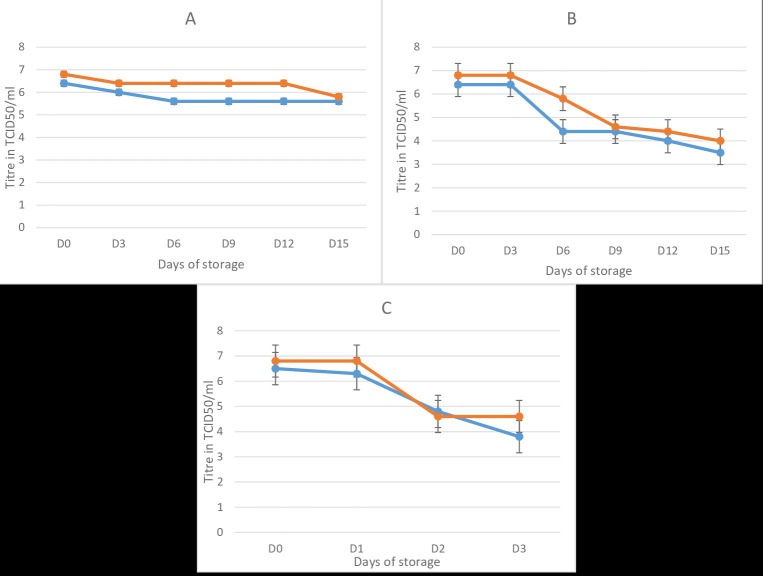
Temperature stability of the RVF CL13T and MP-12NSm-del in lyophilized form at 24°C (A) 37°C (B) and 45°C (C). In bleu, the stability of RVF CL13T vaccine, in red the RVF MP-12 Nsm-del vaccine. D: days

The freeze-dried RVFV vaccine viruses CL13T and arMP-12ΔNSm21/384 remained stable at 4°C for more than 18 months (data not shown). At room temperature (24°C),the infectivity titers for both viruses decreased by 0.4 logs after 3 days of storage, then the titer remained stable during 15 days for CL13T and 12 days for MP-12NSm-del. At 37°C, the titer of the freeze-dried vaccines decreased from 106.4 to 103.5 TCID50/mL for CL13T and from 106.8 to 104.0 TCID50/mL for the arMP-12ΔNSm21/384. At 45°C, the CL13T showed a decrease of 2.8 logs TCID50, and the arMP-12ΔNSm21/384 decreased by 3.3 log TCID50 in 4 days (Figure 1).

**Figure 2:**
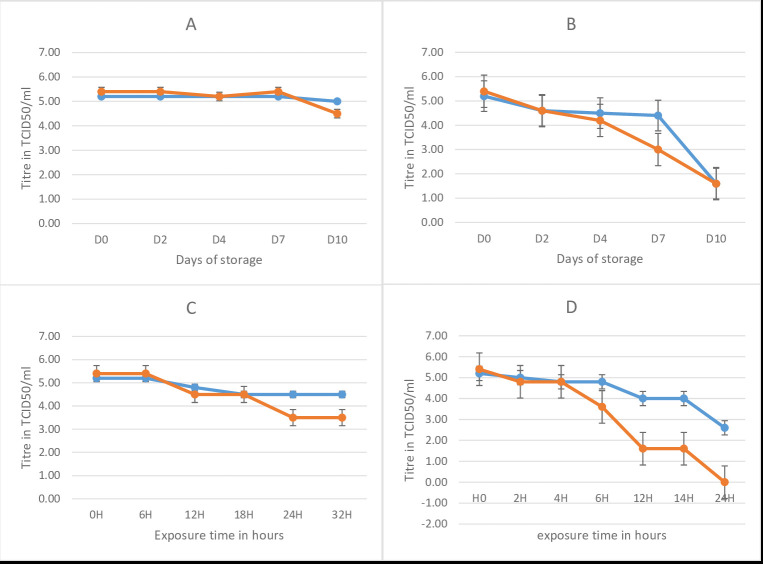
Temperature stability of the RVF CL13T and MP-12N Sm-del after reconstitution in diluent at 4°C (A), 24°C (B) 377deg;C (C) and 45°C (D). In bleu, the stability of RVF CL13T vaccine, in red the RVF MP-12Nsm-del vaccine. H: hours.

After reconstitution in the diluent at 4°C, the CL13T and arMP-12ΔNSm21/384 were stable for 10 days and, 7 days respectively. At room temperature, the CL13T lost 0.8 log in viral titer whereas the arMP-12ΔNSm21/384 lost 2.8 logs in 4 days. At 37°C, the titer of CL13T dropped by 0.4 log (from 105.2 to 104.8) in the first 12 hours and then stabilized at 104.5 TCID50 for more than 32 hours. For the same duration, the arMP-12ΔNSm21/384 titer decreased from 5.4 to 3.5 logs. At 45°C, the CL13T remained stable for more than 3 hours, then the virus titer dropped 2.2 log after 32 hours of exposure, during which time the arMP-12ΔNSm21/384 loss all infectivity (Figure 2). This study provided evidence that both vaccine viruses were more thermos-stable in the freeze-dried form (15 days at 37°C) or liquid form (32 hours at 37°C), with the CL13T being more stable than there combinant arMP-12ΔNSm21/384 vaccine virus (Table 1).

## Discussion:

No significant difference was observed between the decrease in infectivity titer of the RVFV CL13T and arMP-12ΔNSm21/384 vaccine viruses at 4°C and room temperature for both liquid and lyophilized forms and at 37°C for the lyophilized form. However, a significant difference (p<0.05) was found between the decrease in titer of the RVF CL13T and arMP-12ΔNSm21/384 at 45°C (liquid and lyophilized forms) and at 37°C (liquid form). The decrease in virus titer after lyophilization, the storage conditions and/or an incapability to preserve the cold chain during the vaccine delivery are problems facing the use of live vaccines. A formalin-inactivated vaccine, commercially available from the Onderspoort Veterinary Institute in South Africa for veterinary use, was reported to be more stable than the live attenuated form of this vaccine [15],Studies showed that the inactivated RVFV vaccine retained its immunogenicity in cattle after storing the vaccine at 4°C or 25°C, or alternating the vaccine storage between 4 and 25°C [16]. The inactivated vaccine is expensive and less efficient for disease control compared to live attenuated vaccines, additionally the inactivated vaccine needs two vaccinations, followed by an annual booster [17,18].

The currently available CL13 attenuated vaccine has been shown to be effective in sheep, goats and cattle [19],but is not recommended in tropical countries as it is unstable at room temperature and at 37°C in both lyophilized and after reconstitution in the diluent [6]. C13T strain was made thermostable by cloning and selection of thermostable particles through three cycles of heating 30 hours at 56°C for every cycle. The most resistant particles have been selected, cloned, purified and amplified in Vero cells, then the vaccine has been produced and tested at different temperatures in liquid and lyophilized forms [8]. In this study, CL13T was stable for a longer time at temperatures (+4°C, 25°C, 37°C, 457deg;C and 56°C) compared to the original strain (CL13) in both lyophilized and reconstituted form [6]. On the other hand, the Smithburn strain, used for the production of the live attenuated vaccine, has significant limitations not only for its teratogenic effect and abortion in pregnant sheep but also for its instability when exposed to 37.5°C for 5 days [20,21], while the two vaccines used in our study remain stable for more than 15 days at the same temperature. Another live attenuated vaccine used in the United States is the RVFV MP12 vaccine, which was derived from the virulent RVFV ZH548 virus, and its genome encodes 4, 9 and 10 mutations in the S, M and L genome segments, respectively, This infectivity of the vaccine virus was reported to be stable in lyophilized form when the cold chain was maintained at -30°C [22].

## Conclusions:

Our analysis shows that both the CL13T and recombinant arMP-12ΔNSm21/384 vaccine viruses were stable for more than 18 months at 47deg;C. We also show that at room temperatures (37°C and 45°C) the CL13T was less temperature sensitive than arMP-12ΔNSm21/384 in both lyophilized and liquid form. These findings are useful for the preparation of RVF vaccines that will avoid the need for a cold chain and therefore, will improve the application of the vaccines under field conditions.

## Figures and Tables

**Table 1:** Summary of the decrease in infectivity titer of freeze-dried and liquid form RVF CL13T and arMP-12ΔNSm21/384 vaccine viruses

Decrease in titer of freeze-dried vaccine viruses expressed by TCID50	Decrease in titer of liquid form of the vaccine viruses expressed by TCID50
Exposure temperature	CL13T	arMP-12ΔNSm21/384	CL13T	arMP-12ΔNSm21/384
24°C	0.8	1	3.6	3.8
37°C	2.9	2.8	0.7	1.9
45°C	2.8	3.3	2.6	5.4
